# Effects of traditional Chinese medicine collapse stains therapy combined with burn ointment on wound healing, inflammation, and pain mediator levels in patients with second degree burns

**DOI:** 10.12669/pjms.41.6.10590

**Published:** 2025-06

**Authors:** Junhui Huang, Hongjuan Liu, Qian Wang, Jiancheng Wang, Xiaocheng Liu

**Affiliations:** 1 Junhui Huang Department of General Surgery, Xingtai Third Hospital, Xingtai 054000, Hebei, China.; 2 Hongjuan Liu Department of Radiology, Hengshui Third People’s Hospital, Hengshui 053099, Hebei, China.; 3 Qian Wang Department of Dermatology, Dingzhou Maternal and Child Health Hospital, Dingzhou 073000, Hebei, China.; 4 Jiancheng Wang Department of Colorectal Surgery, Nanpi County People’s Hospital, Cangzhou 061599, Hebei, China; 5 Xiaocheng Liu Department of Infection, Cangzhou Hospital of Integrated TCM-WM in Hebei, Cangzhou 061013, Hebei, China

**Keywords:** Inflammation, Pain Mediators, Second-Degree Burns, Traditional Chinese Medicine Collapse Stains Therapy, Wound Healing

## Abstract

**Objective:**

To investigate the effects of traditional Chinese medicine(TCM) collapse stains therapy combined with burn ointment on wound healing, inflammation, and pain mediator levels in patients with second-degree burns.

**Methods:**

This was a retrospective study. A total of 84 patients with second degree burns treated at Xingtai Third Hospital from January 2022 to December 2023 were selected as the study subjects. They were randomly divided into a control group (treated with burn ointment) and a combined treatment group (received both TCM collapse stains therapy and burn ointment). The two groups were compared in terms of wound healing, inflammation, complication rates, and patient satisfaction to comprehensively evaluate the treatment effects.

**Results:**

Before treatment, there was no statistically significant difference in the indicators between the two groups (p>0.05). After three treatment courses, the wound healing rate in the combined group (83.33%) was higher than that in the control group(64.29%). The levels of inflammatory factors and pain mediators in the combined group were lower than those in the control group. The complication rate in the combined group(14.29%) was significantly lower than that in the control group (33.33%), and the satisfaction rate of the combined group(90.48%) was significantly higher than that of the control group(73.81%), with significant differences in all indicators(p<0.05).

**Conclusion:**

The application of TCM collapse stains therapy combined with burn ointment may have a significant effect on the treatment of second-degree burns. It may effectively promote wound healing, reduce levels of inflammatory factors and pain mediators, lower complication rates, and increase patient satisfaction.

## INTRODUCTION

Burns are characterized by skin or mucosal tissue damage caused by heat, and are classified into various types, including flame burns, chemical burns, and electrical burns. These injuries pose a significant global public health challenge.^1^,^2^ Second-degree burns, which penetrate into the dermal layer, are a prevalent category of burns. The quality of life for patients suffering from second-degree burns is considerably compromised due to pain, financial burden of treatment, and functional impairment.^3^-^5^ Consequently, exploring the mechanisms underlying burn wound healing and the development of efficacious therapeutic strategies to promote the healing of second-degree burns are of critical importance.

Early and effective treatment of burn wounds is crucial for the management of second-degree burns and is closely related to the patient’s post-recovery appearance, duration of illness, complications, and functional recovery.^6^ With the increase in antibiotic resistance and adverse reactions to Western medicines, burn healing professionals have attached greater importance to the use and development of traditional Chinese medicine in recent years.^7^,^8^ Chinese herbal burn ointments help to reduce inflammation and pain, activate blood circulation to dissipate blood stasis, and promote the proliferation of fibroblasts and keratinocytes in the wound, thus treating both the root cause and symptoms.^9^,^10^ TCM collapse stains therapy can alleviate local pain and inflammation through moist dressing conduction and radiation effects. It is widely used, easy to operate, and safe and painless. Currently, it has achieved remarkable outcomes in the nursing of osteoarthritis, muscle strain, and traumatic injuries.^11^ Therefore, this study analyzed the effects of TCM collapse stains therapy combined with burn ointment on wound healing, inflammation, and pain mediator levels in patients with second-degree burns.

## METHODS

This was a retrospective study. A total of 84 Chinese patients with second degree burns admitted to Xingtai Third Hospital from January 2022 to December 2023 were selected as the study subjects. According to the treatment method, the patients were divided into a control group and a combination therapy group, with 42 patients in each group. The control group comprised an equal number of males and females, 21 of each gender, with an average age of (35.21±12.89) years, ranging from 15 to 55 years old. Similarly, the combination therapy group consisted of 21 males and 21 females, with an average age of (37.31±12.43) years, spanning from 14 to 56 years old. The comparison of general information between the two groups of patients with second-degree burns revealed no statistically significant difference (p>0.05).

### Ethics approval:

The study was approved by the Institutional Ethics Committee of Xingtai Third Hospital (No: 2022643; Date: April 15, 2023), and written informed consent was obtained from all participants’ guardians.

### Inclusion criteria:

- Patients diagnosed with second-degree burns according to diagnostic criteria after admission.
- Able to communicate normally and strictly follow the nursing requirements for treatment.
- Patients and their families voluntarily participated in this research project and signed relevant documents.
- Patients undergoing treatment for second-degree burns for the first time.

### Exclusion criteria:

- Patients with mental or psychological disorders or unable to communicate normally.
- Patients with malignant tumors, severe organ diseases, systemic diseases, etc.
- Patients in the preconception period, pregnancy, or lactation.
- Patients allergic or contraindicated to the medications used in this study.

### Control Group:

Patients received burn ointment treatment. Following cleaning and disinfection of the local skin, the burn ointment was applied to both the injury site and adjacent skin areas. This application was repeated at four-hour intervals to maintain a moist environment conducive to healing until the eschar naturally sloughed off, signaling recovery. Nursing staff provided comprehensive education to both patients and their families regarding the critical precautions associated with the use of burn ointment, emphasizing the importance of preventing infection and avoiding any actions that might disrupt the integrity of the wound and impede the healing process.

### Combination therapy group:

Patients with second-degree burns were treated with TCM collapse stains therapy combined with burn ointment. The burn ointment treatment method was the same as that in the control group. The formula for traditional Chinese medicine collapse stains therapy included: 10 g of Honeysuckle, 10 g of Giant Knotweed, 10 g of Notoginseng Root, 10 g of Kirilow Rhodiola Root and Rhizome, and 5 g of Chinese Goldthread Rhizome.^12^ The ingredients were encased in a single-use cloth pouch, which was then thoroughly saturated with purified water and steamed for 8-10 minutes. After the cleaning and disinfection of the affected area and adjacent skin, the cloth pouch was applied to the affected area with a thickness of about 1cm. At the same time, an intermediate-frequency pulse therapy device was employed with meticulous attention to avoid thermal injury to the patient. Each treatment session spanned 30 minutes, with a complete course of therapy extending over seven days and the entire regimen encompassing a total of five treatment courses. Additionally, routine nursing education was provided to patients and their families to avert any daily care practices that could detrimentally affect therapeutic outcomes.

### Observation indicators:

- *(1) Comparison of wound healing efficacy:* The healing efficacy of wounds in both patient groups was evaluated after three treatment courses, with outcomes classified as cured, markedly effective, effective, and ineffective. The total effective rate was calculated. Total effective rate = (cured + markedly effective + effective) / total number of cases * 100%.
- *(2) Comparison of inflammatory factor levels:* The levels of inflammatory factors in the serum of the two groups of patients before and after treatment were measured. Enzyme-linked immunosorbent assay was used to determine the levels of tumor necrosis factor-α(TNF-α), interleukin-6(IL-6), and interleukin-10(IL-10) in the serum.
- *(3) Comparison of pain mediator levels:* The levels of pain mediators in the serum of patients in both groups before and after treatment were measured. Enzyme-linked immunosorbent assay was used to determine the levels of 5-hydroxytryptamine (5-HT), neuropeptide Y (NPY), and prostaglandin E2 (PGE2) in the serum.
- *(4) Comparison of complication rates:* The incidence of various complications, including wound infection, edema, joint spasm, vascular spasm, and bacteremia were counted in the two groups of patients, and the total incidence was calculated. Total incidence rate = (wound infection + edema + joint spasm + vascular spasm + bacteremia) / total number of cases * 100%.
- *(5) Patient satisfaction:* The medical team reviewed relevant literature on China National Knowledge Infrastructure(CNKI) and designed a patient satisfaction questionnaire. The survey encompassed multiple dimensions such as satisfaction with the treatment regimen, nurses’ communication proficiency, sense of responsibility, and the timeliness and effectiveness of problem resolution, with a total score of 100. Scores below 60 were deemed unsatisfactory, scores between 60 to 79 were rated as average, and scores of 80 or higher were considered satisfactory. The satisfaction rate was the aggregate of satisfactory and average scores. For patients aged under 18, the questionnaires were collaboratively completed by both the patients and their guardians.

### Statistical analysis:

SPSS 22.0 statistical software was used for data processing and analysis. The confidence interval was 95%. Measurement data were expressed as (*x̄* ± *s*), and the t-test was used to compare measurement data. The *χ^2^* test was used to compare count data. Count data were expressed as percentages (%). p<0.05 was considered statistically significant.

## RESULTS

As shown in Table-I, the wound healing rate in the combination therapy group(83.33%) was significantly higher than that in the control group(64.29%), and the difference was statistically significant(p<0.05). As shown in Table-II, before treatment, there was no statistically significant difference in TNF-α, IL-6, and IL-10 levels between the two groups of patients (p>0.05). After treatment, the scores of TNF-α, IL-6, and IL-10 in the combination therapy group were lower than those in the control group, and the differences were statistically significant (p<0.01).

**Table-I:** Comparison of wound healing effects between the two groups of patients (n=42).

Group	Cured (n%)	Markedly effective (n%)	Effective (n%)	Ineffective (n%)	Wound healing rate (%)
Combination therapy group	8(19.05)	11(26.19)	16(38.10)	7(16.67)	83.33
Control group	5(11.90)	6(14.29)	15(35.71)	16(38.10)	64.29
*χ^2^*	-	-	-	-	4.850
*P*	-	-	-	-	0.028

**Table-II:** Comparison of inflammatory factor levels between the two groups of patients (n=42, x̄±s).

Quality of life	TNF-α(μg/L)		IL-6(pg/mL)		IL-10(pg/mL)	
	Before treatment	After treatment	Before treatment	After treatment	Before treatment	After treatment
Combination therapy group	6.95±0.54	3.32±0.32	141.31±6.88	110.26±6.38	37.45±4.86	22.05±3.89
Control group	6.87±0.55	5.04±0.35	142.62±6.97	137.69±8.22	37.52±5.09	26.93±3.05
*t*	0.637	-23.367	-0.867	-17.083	-0.066	-6.396
*P*	0.526	5.288E-38	0.388	5.863E-28	0.948	1.103E-8

Before treatment, there was no statistically significant difference in 5-HT, NPY, and PGE2 levels between the two groups of patients (p>0.05). Table-III. After treatment, the levels of 5-HT, NPY, and PGE2 in the combination therapy group were lower than those in the control group, and the differences were statistically significant (p<0.01).

**Table-III:** Comparison of pain mediator levels (n=42, x̄±s).

Quality of life	5-HT(ng/L)		NPY(μg/L)		PGE2(ng/L)	
	Before treatment	After treatment	Before treatment	After treatment	Before treatment	After treatment
Combination therapy group	220.45±12.65	85.38±10.63	226.38±14.39	133.60±8.88	224.38±13.84	126.26±11.96
Control group	219.95±12.37	130.62±7.33	222.43±14.79	171.45±12.99	224.07±15.64	184.98±7.95
*t*	0.183	-22.702	1.241	-15.590	0.096	-26.507
*P*	0.855	9.430E-35	0.218	7.576E-25	0.924	1.179E-38

The complication rate in the combination therapy group (14.29%) was significantly lower than that in the control group (33.33%), and the difference was statistically significant (p<0.05). Table-IV. After the treatment experiment, the satisfaction rate in the combination therapy group (90.48%) was significantly higher than that in the control group (73.81%), and the difference was statistically significant (p<0.05). Table-V.

**Table-IV:** Comparison of complication rates between the two groups of patients.

Group	Wound infection	Edema	Joint spasm	Vascular spasm	Bacteremia	Total incidence rate (n%)
Combination therapy group	2	2	1	1	0	6(14.29)
Control group	4	4	1	3	2	14(33.33)
*χ^2^*	-	-	-	-	-	4.200
*P*	-	-	-	-	-	0.040

**Table-V:** Comparison of patient satisfaction (n=42).

Complications	Unsatisfactory	Average	Satisfactory	Satisfaction rate (n%)
Combination therapy group	4	6	32	38(90.48)
Control group	11	11	20	31(73.81)
*χ^2^*	-	-	-	3.977
*P*	-	-	-	0.046

## DISCUSSION

This study found that the wound healing rate in the combination therapy group (83.33%) was higher than that in the control group (64.29%), and the complication rate in the combination therapy group (14.29%) was significantly lower than that in the control group (33.33%). The combination therapy group exhibited more favorable outcomes, and the levels of inflammatory factors and pain mediators were lower than those in the control group, effectively alleviating patients’ discomfort during the treatment process. The satisfaction rate in the combination therapy group (90.48%) was significantly higher than that in the control group (73.81%). The differences in various indicators were statistically significant (p<0.05). Burn wounds elicit a spectrum of inflammatory responses within the organism^13^,^14^, These physiological challenges can precipitate multiple organ failure. Beyond the immediate physical health implications, burn injuries can profoundly affect the psychological well-being and overall quality of life of patients.^15^ Wound healing is a complex, dynamic pathophysiological process encompassing hemostasis, inflammation, re-epithelialization, and tissue remodeling phases. Disruptions in any phase can impede healing, potentially leading to exacerbated wound conditions, extended recovery periods, and the onset of complications.^16^-^18^ The inflammatory and re-epithelialization phases, in particular, are critical determinants of wound healing velocity. Triggering the release of pro-inflammatory cytokines, notably tumor necrosis factor-alpha (TNF-α), interleukin-1 (IL-1), and interleukin-6 (IL-6). Pain mediators such as serotonin (5-HT), neuropeptide Y (NPY), and prostaglandin E2(PGE2) play pivotal roles in this context. Enhances 5-HT platelet production, leading to local edema and an amplified pain experience. NPY contributes to cell membrane depolarization and increased vascular excitability, thereby intensifying pain perception. PGE2 facilitates the inflammatory response and expedites pain signal transmission along neural pathways. Burn ointments serve to occlude wounds, prevent infection, and exhibit antibacterial and anti-inflammatory properties. When combined with medium-frequency pulse therapy, TCM collapse stains can induce vasodilation, while the herbal constituents offer anti-inflammatory, analgesic, and blood stasis-resolving benefits, mitigating inflammation and pain at the wound site.^19^,^20^ Pre-treatment comparisons revealed no significant statistical differences in the evaluated indicators between the two groups (p>0.05). After three treatment courses, the combination therapy group exhibited lower levels of inflammatory factors (TNF-α, IL-6, IL-10) and pain mediators (5-HT, NPY, PGE2) compared to the control group.

### Limitations

TCM collapse stains therapy involves multiple, intricate steps, which may pose adherence challenges for patients with mobility impairments, potentially compromising therapeutic effectiveness. Given that TCM is typically administered through simple steaming with a brief application duration, there is a notable loss of medicinal efficacy and a consequent financial burden on patients. Further research is needed to explore the transformation of TCM collapse stains pouch into paste patches, with the aim of streamlining the application process, optimizing drug utilization, improving patient compliance, and ultimately reducing the cost of treatment.

## CONCLUSIONS

The application of TCM collapse stains therapy combined with burn ointment may have a significant therapeutic effect on second-degree burns, effectively promoting wound healing, reducing the levels of inflammatory factors and pain mediators, lowering the complication rate, and improving patient satisfaction.

### Authors’ Contributions:

**JH** and **HL:** Carried out the studies, participated in collecting data and drafted the manuscript and are responsible and accountable for the accuracy or integrity of the work.

**QW** and **JW:** Collected and analyzed clinical data. Critical Review.

**XL:** Performed the statistical analysis and participated in its design.

All authors have read and approved the final manuscript.
